# CRISPR/Cas9 Genome Editing vs. Over-Expression for Fluorescent Extracellular Vesicle-Labeling: A Quantitative Analysis

**DOI:** 10.3390/ijms23010282

**Published:** 2021-12-28

**Authors:** Karin Strohmeier, Martina Hofmann, Fabian Hauser, Dmitry Sivun, Sujitha Puthukodan, Andreas Karner, Georg Sandner, Pol-Edern Le Renard, Jaroslaw Jacak, Mario Mairhofer

**Affiliations:** 1 Department of Medical Engineering and Applied Social Sciences, University of Applied Sciences Upper Austria, Garnisonstraße 21, 4020 Linz, Austria; karin.strohmeier@fh-linz.at (K.S.); martina.hofmann@fh-linz.at (M.H.); fabian.hauser@fh-linz.at (F.H.); dmitry.sivun@fh-linz.at (D.S.); sujitha.puthukodan@fh-linz.at (S.P.); andreas.karner@fh-linz.at (A.K.); jaroslaw.jacak@fh-linz.at (J.J.); 2 Center of Excellence Food Technology and Nutrition, University of Applied Sciences Upper Austria, Stelzhamerstraße 23, 4600 Wels, Austria; georg.sandner@fh-wels.at; 3 Center of Advanced Bioanalysis GmbH, Gruberstraße 38, 4020 Linz, Austria; lerenard.pe@gmail.com; 4 Austrian Cluster for Tissue Regeneration, 1200 Vienna, Austria

**Keywords:** extracellular vesicles, genome editing, CRISPR/Cas9, single-molecule fluorescence microscopy, atomic force microscopy, single-molecule labeling stoichiometry, CD63

## Abstract

Over-expression of fluorescently-labeled markers for extracellular vesicles is frequently used to visualize vesicle up-take and transport. EVs that are labeled by over-expression show considerable heterogeneity regarding the number of fluorophores on single particles, which could potentially bias tracking and up-take studies in favor of more strongly-labeled particles. To avoid the potential artefacts that are caused by over-expression, we developed a genome editing approach for the fluorescent labeling of the extracellular vesicle marker CD63 with green fluorescent protein using the CRISPR/Cas9 technology. Using single-molecule sensitive fluorescence microscopy, we quantitatively compared the degree of labeling of secreted small extracellular vesicles from conventional over-expression and the CRISPR/Cas9 approach with true single-particle measurements. With our analysis, we can demonstrate a larger fraction of single-GFP-labeled EVs in the EVs that were isolated from CRISPR/Cas9-modified cells (83%) compared to EVs that were isolated from GFP-CD63 over-expressing cells (36%). Despite only single-GFP-labeling, CRISPR-EVs can be detected and discriminated from auto-fluorescence after their up-take into cells. To demonstrate the flexibility of the CRISPR/Cas9 genome editing method, we fluorescently labeled EVs using the HaloTag^®^ with lipid membrane permeable dye, JaneliaFluor^®^ 646, which allowed us to perform 3D-localization microscopy of single EVs taken up by the cultured cells.

## 1. Introduction

A myriad of cell types, including epithelial, endothelial, blood, stem, and tumor cells release small vesicles into their extracellular milieu [1,2,3,4]. These vesicles contain transmembrane and soluble proteins [5,6,7], RNA [8], and DNA [9] molecules, which are membrane-enclosed and protected from extracellular proteases and nucleases. Due to these characteristics, they are ideal candidates for diagnostic and drug-delivery applications [10,11,12,13,14,15,16].

Extracellular vesicles (EVs) are a heterogenous family that are basically defined by their biogenesis, size, and composition. The most extensively investigated subtypes of extracellular vesicles are endosome-origin exosomes, which are derived from multi-vesicular bodies, and plasma membrane-derived ectosomes (or microvesicles) which directly bud from the cell membrane [17,18]. Exosomes typically have a diameter of 30–100 nm [3,19] and are strongly enriched in membrane proteins such as CD9, CD81, and CD63 [20,21]. These proteins, therefore, are widely employed as exosome markers. 

Despite extensive research, many fundamental questions concerning the biogenesis, cargo sorting, secretion, transport, and uptake of EVs by recipient cells remain unanswered. Imaging, dynamic tracing, and the characterization of individual EVs have recently been performed with the help of fluorescence microscopy [22,23,24] and atomic-force microscopy [25,26]. Single-molecule fluorescence microscopy (SMFM) is a versatile technique that enables the visualization of intracellular distribution and dynamics of EVs [27,28,29]. Nevertheless, it relies on the efficient and stable labeling of the investigated structures with fluorophores. Previously, for EV imaging, the over-expression of FP-tagged marker proteins [29,30], click-chemistry mediated [31], immune [32], or nonspecific organic dye-based staining has been used for fluorescence labeling [33].

To date, many studies have relied on the transient or stable cellular over-expression of fluorescently-tagged fusion proteins [23,24,29,34]. Over-expression enhances the fluorescent signal but can cause a disturbance of the endogenous stoichiometric balance of interacting cellular components, ectopic cellular localizations, and self-association of fluorophore tags, leading to abnormal cellular phenotypes [34,35] and can also lead to an uneven loading of the proteins in EV membranes and can change their properties [21,22,23]. 

Therefore, expression levels that are closest to the physiological state are preferred above over-expression systems [36,37]. To avoid artefacts that are caused by over-expression and to enable a more native level of the labeled protein, we used the genome-editing technology, CRISPR/Cas9, and compared it to the conventional over-expression method. The clustered regularly interspaced short palindromic repeat (CRISPR) and CRISPR-associated protein 9 (Cas9) system [38] revolutionized the field of genetic engineering [39,40,41]. This was achieved through its facilitating simple and efficient editing of almost any desired target genomic locus. Through provision of a repair plasmid, carrying the desired transgene, the cellular homology-directed repair (HDR) pathway is exploited to insert the DNA sequence of a fluorescent protein (FP), in-frame, with the protein of interest. The fluorescent fusion protein enables the visualization of the intracellular localization dynamics of the target protein under endogenous regulatory control [37,42,43]. Consequently, this method has been used for single-molecule-based nanoscopic imaging of living cells [44,45]. To the best of our knowledge, CRISPR/Cas9 technology has been never used for single-molecule studies of EVs despite all the merits. 

Another widely used labeling option for cellular imaging is the HaloTag^®^ technology. The HaloTag^®^ is a modified bacterial haloalkane dehalogenase that was designed to form a covalent bond between the protein and the chloroalkane linker of synthetic ligands, providing a flexible labeling method [46,47]. The available membrane-permeable dyes provide a better photon yield compared to a single GFP fluorophore and show high photostability and high signal–noise ratio, which is promising when performing live-cell imaging and particle tracking [47,48,49]. 

In this study, we tagged the N-terminus of the EV marker protein CD63 with a green fluorescent protein (GFP) as well as with the HaloTag^®^ using the CRISPR/Cas9 system. We isolated EVs from the targeted cells and analyzed their properties regarding size, labeling density, and intracellular uptake at the single-molecule level. The CRISPR/Cas9 GFP-CD63-labeled EVs were compared with EVs that were labeled by over-expression of GFP-CD63. Precisely, using SMFM and AFM, we were able to show that despite comparable size distributions, the EVs that were isolated from the CRISPR/Cas9 transfected cells carried predominantly one GFP molecule per vesicle. In contrast, the EVs that were derived from the over-expressed cells carried a significantly higher amount of GFP molecules. By employing single-molecule localization microscopy, we performed intracellular imaging of uptake and analysis of EVs distributions inside the cells. 

The CRISPR/Cas9-modified EVs carrying GFP-CD63 or HaloTag^®^-CD63 provide a potent model system for studying the cellular transport and uptake mechanisms of EVs in living cells. The reduced labeling stoichiometry that is obtained in the CRISPR/Cas9-modified EVs outperforms the highly loaded EVs with over-expressed GFPs. An improved quantitative analysis of the localization, distribution, and uptake of individual EVs inside the cells can be provided. The combination of the more homogeneous labeling by genome editing and the ability to use bright and photostable organic dyes that are compatible with the HaloTag^®^ opens new possibilities for investigating biogenesis, transport, and uptake of single EVs in both in vitro and in vivo models.

## 2. Results

### 2.1. CRISPR/Cas9 Fluorescent Labeling of EVs 

**Figure 1:**
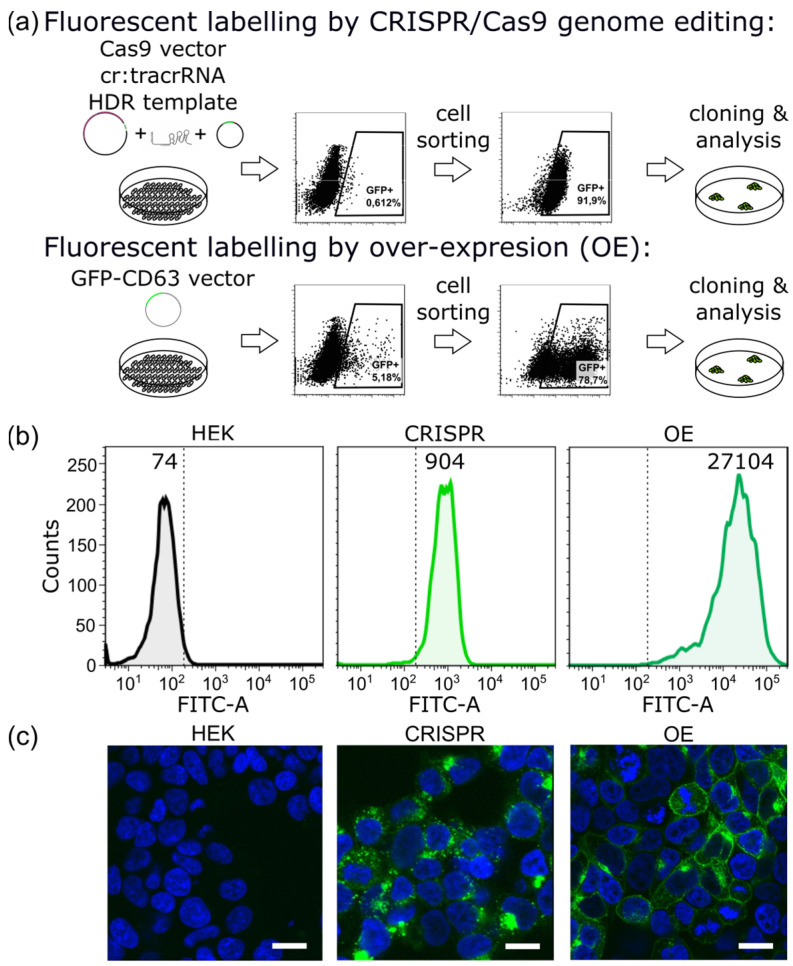
CRISPR/Cas9 approach and over-expression approach for fluorescent labeling of the EV marker CD63. (**a**) A schematic representation of the HEK293T cell transfections. The cells were either transfected with a Cas9 expression vector together with a guideRNA targeting exon 2 of CD63 and a homology-dependent repair (HDR) template encoding GFP in-frame with the CD63 coding sequence (upper panel) or with a vector encoding a GFP-CD63 fusion under the control of the CMV promoter (lower panel). After transfection, GFP fluorescence was detected in a subset of the cells, which were enriched by FACS sorting. In the next step, single-cell-derived clones were isolated and characterized by flow cytometry and immunofluorescence. (**b**) Flow cytometry analysis shows median fluorescence intensity (MFI) of GFP expression that was acquired in FITC-A channel. The parental HEK293T cells (HEK) are included as a negative control compared to the CRISPR/Cas9-edited GFP-CD63 HEK293T cells (CRISPR) and cells over-expressing GFP-CD63 (OE). (**c**) Untransfected HEK cells, CRISPR-, and OE-clones were seeded onto glass slides, fixed, counter-stained with DAPI, and analyzed with an Olympus FV10i confocal microscope. The CRISPR clone displays perinuclear GFP fluorescence, as expected for CD63, with minimal plasma membrane localization. In the OE clone, the plasma membrane localization of GFP-CD63 is clearly detected in a large fraction of the cells. The scale bar is 10 µm.

To fluorescently label EVs, the over-expression of an FP-tagged CD63 protein was used by several groups to study the uptake and sub-cellular transport in recipient cells [50,51,52]. Strong over-expression, although desirable to achieve intense g of EVs, bears the risk of substantially changing their protein composition and, thereby, also their properties [21,22,23]. Fluorescent-tagging of the endogenous CD63 protein utilizing the CRISPR/Cas9 genome editing technique [38,39] might avoid these restrictions. We, therefore, performed a gene knock-in of enhanced GFP to the N-terminus of CD63 in HEK293T cells via the CRISPR/Cas9 system, leading to an intra-vesicular localization of the GFP moiety, and isolated EVs from this genome-edited stable cell line (designated as “CRISPR-EV”). For comparison, EVs were isolated from an over-expressed GFP-CD63 cell line (designated as “OE-EV”). Figure 1a summarizes the protocol for transfection, cell sorting, and cloning of the GFP-positive cells for both transfection methods. To demonstrate the integration of GFP in the genome via CRISPR/Cas9, we performed an integration-specific PCR analysis from genomic DNA that was isolated from multiple GFP+ sorted cell clones. We used a GFP-specific forward primer and a CD63-specific reverse primer binding outside of the CD63 homology arm that was cloned into the HDR plasmid. All the isolated CRISPR/Cas9 clones showed correct integration of GFP at the endogenous CD63 locus, as demonstrated by the 1748 bp product, which is absent from parental HEK293T cells (Supplementary Figure S1a). A second PCR using a CD63-specific forward primer up-stream of the GFP integration site, and the CD63-specific reverse primer demonstrated that, most likely, a single CD63 allele was targeted in all clones, as the wild-type PCR product of 1203 bp was detectable together with the 1932 bp product including GFP (Supplementary Figure S1a). Thus, the PCR revealed that, most likely, GFP was integrated at only one of the CD63 alleles in the HEK293T cells. The GFP expression levels in the CRISPR and the OE cell clones were compared by flow cytometry measurements. Figure 1b shows the median fluorescence intensity (MFI) of GFP for parental HEK cells, CRISPR, and OE-modified cell lines. MFI was increased 12-fold for CRISPR and 366-fold for OE-modified clones, respectively, compared to the parental cell line.

For better understanding of the sub-cellular localization of GFP-tagged CD63 inside the CRISPR and OE cells, we have performed confocal imaging of the modified and control HEK cells (Figure 1c). The over-expression of CD63 had a profound effect on the subcellular localization of the protein, leading to an increase of CD63 at the plasma membrane in a large fraction of the cells. In contrast, the CRISPR clone displayed a perinuclear GFP distribution, as expected for CD63, with minimal plasma membrane localization. 

**Figure 2:**
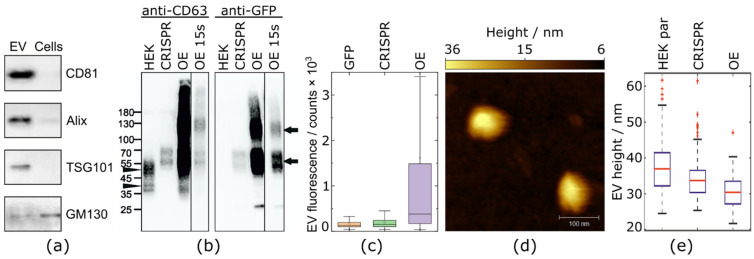
The characterization of extracellular vesicles and the expression of GFP-CD63. The EVs were enriched from either parental HEK293T cells or cells that were stably expressing GFP-CD63—achieved either by CRISPR/Cas9 (CRISPR-EV) or a conventional over-expression system (OE-EV). (**a**) Small EVs that were isolated from CRISPR GFP-CD63 stable cell lines were compared to total cell lysates by Western blotting of positive marker proteins for EVs (CD81, Alix, TSG101) and the negative marker GM130. (**b**) Comparison of EVs from parental HEK293T cells, CRISPR-EVs, and OE-EVs. Equal amounts (5 µg) of total protein were loaded and the membrane was probed either with an anti-CD63 antibody or an anti-GFP antibody. The bands for endogenous CD63 (arrowheads, left side) and for the GFP-CD63 fusion proteins (arrows, right side) are indicated. A 15-s short-time exposure of the OE-samples was added to enable a comparison of the band sizes to the other samples. (**c**) Recombinant GFP protein, CRISPR-EVs, and OE-EVs were immobilized on Poly-D-Lysine (PDL)-coated glass cover slips. The intensity profiles of the fluorescent spots were fit with a Gaussian function and the corresponding distribution fits of the integrated intensities are shown as box plots. (**d**) HS-AFM image of isolated EVs, exemplarily shown for CRISPR-EVs. The color bar expresses the height of the vesicles in nm. (**e**) HS-AFM analysis EVs of parental HEK293T, CRISPR-EVs, and OE-EVs. The particle height was measured as described in the Materials & Methods. Approximately 70 measurements per sample are depicted in the box plot, the median is indicated by the red line.

Next, we have isolated EVs from both the modified cell lines by sequential centrifugations as adapted from Bobrie et al. [53]. The cellular debris, apoptotic bodies, and larger EVs were removed from the supernatants by centrifugation. According to the Minimal Information for Studies of Extracellular Vesicles (“MISEV2018”) guidelines [17], our isolated EVs are classified as the so-called small EV-fraction. For simplicity reasons, we will use the general term EVs hereafter. Western blot analysis, as shown in Figure 2a, evidenced a significant enrichment of common EV marker proteins (CD81, Alix, TSG101) and the absence of the Golgi protein, GM130, indicating adequate purity of the preparations. 

To confirm the GFP-CD63-fusion protein, we compared the isolated EVs from culture supernatants of parental HEK293T cells, the CRISPR, and the OE-modified cell line by Western blot. Equal amounts (5 µg) of total protein were loaded and the membrane was probed either with an anti-CD63 antibody or an anti-GFP antibody (Figure 2b). The parental HEK control showed the endogenous CD63 protein fraction. Both the modified cell lines showed a shifted band as confirmation for the GFP-CD63-fusion, with a much broader and brighter band in the OE-EVs compared to CRISPR-EVs. The OE GFP-EVs showed only a small fraction of free GFP while there was no visible fraction of free GFP in the CRISPR-EVs.

To gain information about GFP-labeling of the isolated EVs, we performed fluorescent measurements of individual EVs and GFP molecules. The EVs, as well as the recombinant soluble GFP proteins, were greatly diluted to yield sparse, well-separated signal spots on a glass substrate. The count rate (per fluorescent spot) of the GFP proteins yielded an average signal of 157 ± 65 a.u. (t_ill_ = 5 ms, *n* = 247, I ~ 3.2 kW/cm^2^). The average intensity signal of a single GFP-labeled CRISPR-EV was 195 ± 168 a.u. (t_ill_ = 5 ms, *n* = 392, I ~ 3.2 kW/cm^2^), which was only slightly higher compared to average single-molecule GFP emission. At the same time, the intensity of a single GFP-labeled OE-EV was much larger, on average 1697 ± 2912 a.u. (t_ill_ = 5 ms, *n* = 498, I ~ 3.2 kW/cm^2^). The box chart for all three distributions is shown in Figure 2c. This high difference between the CRISPR and OE EVs is not surprising given the much higher fluorescence of the respective cell lines (Figure 1b,c). The high average intensity, as well as the broadness of the fluorescence intensity distribution for OE-EVs suggests a strong population heterogeneity. To exclude the influence of simple aggregation of the OE-EVs on the fluorescence data, high-speed atomic force microscopy (HS-AFM) was performed. HS-AFM analysis didn’t reveal an aggregation of EVs and indicated their small vesicular character, with a mean size ranging from 30–40 nm. The size distributions (Figure 2e) showed only marginal differences among the EVs originating from the parental HEK293T cells, the CRISPR transfected clones, and the over-expressing clones. The presence of debris, including free GFP molecules as a degradation product of a potentially impaired fusion protein, which could be another reason for much broader fluorescence distribution of OE-EVs, was eliminated by Western blot analyses, as shown in Figure 2b. Only a small fraction of free GFP was detected (27 kDa) in the OE-EV samples.

**Figure 3:**
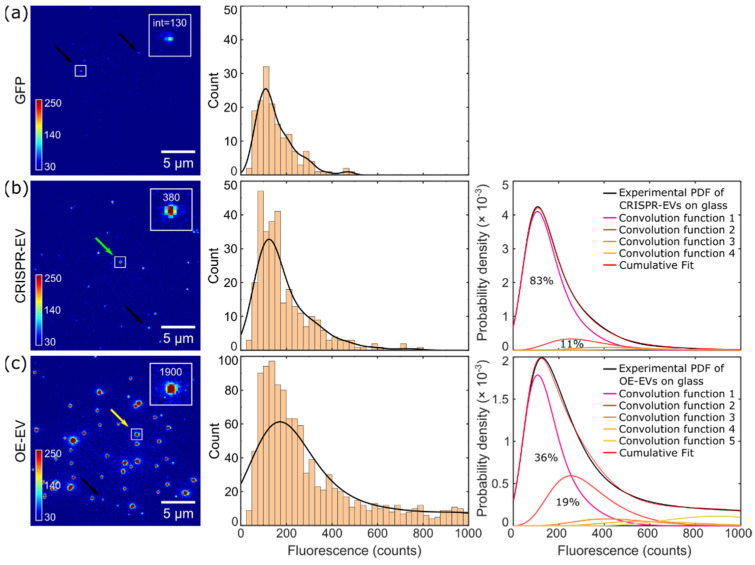
Fluorescence signals of single (**a**) GFPs, (**b**) CRISPR-EVs, and (**c**) OE-EVs that were immobilized on Poly-D-Lysine (PDL)-coated glass cover slips. The spots on the fluorescence image (left column) were fitted with a gaussian and then the integrated intensity for each spot were determined. The middle column shows histograms and the probability density function (PDF) as a black solid line for each population, respectively. The probability densities of GFP-labeled CRISPR-EVs and OE-EVs were convoluted with multiple GFP PDFs (right column). The probability density function convolution of GFP intensities with CRISPR-EVs (83%—DOL = 1, 11%—DOL = 2, 4%—DOL = 3, 2%—DOL ≥ 4) and OE-EVs (36%—DOL = 1, 19%—DOL = 2, 3%—DOL = 3, 42%—DOL ≥ 4), respectively, show that over-expression introduces a higher number of CD63-GFP constructs into the EV membrane. (DOL = degree of labeling). Black arrows indicate typical signals for a single GFP, the green arrow indicates an EV assigned with two GFP labels and the yellow arrow indicates an EV with multiple GFP labels.

To gain a more detailed picture of the extent of GFP-labeling, CRISPR-EVs and OE-EVs were analyzed using SMFM. In detail, we compared the fluorescence distributions of both the isolated EV populations with the distribution of single GFP proteins. Considering the high dilution of GFP, each detected fluorescence spot with high probability represents a single GFP molecule (Figure 3a). Each detected spot was parametrized by 2D Gaussian fitting [54,55,56,57]. By measuring a large number of spots (*n* = 247) one can obtain the distribution of fluorescence intensities (Figure 2c and Figure 3a). The probability density function (PDF) that was obtained from such distribution (black solid lint in Figure 3a) can be used to estimate the probability of having one, two, or more GFP molecules in single EV. PDF that was obtained for single GFP and integer multiples thereof were convoluted with the PDF that was measured for EVs sample. The maxima of each convolution function were fixed at integer multiples of the average single GFP intensity distribution. (Figure 3b,c, right panels). The labeling degree was then directly related to the integrated area of respective convolution [58].

We determined that ~83% of CRISPR-EVs contained a single GFP-labeled CD63. Furthermore, ~11% had two GFP-labeled CD63 proteins, and approximately ~4% carried three proteins. The remaining ~2% were multiple (≥ 4 GFP) labeled EVs. In contrast, in the OE-EV samples only ~36% of all the detected signals showed the properties of a single GFP emitter, ~19% of the population had signals corresponding to two GFP fluorophores, around ~3% of the population had signals corresponding to three GFP fluorophores, and the remaining 42% represented the population of multiple labeled EVs (≥ 4 GFP). These data, in general, indicated that CRISPR-EVs, in most of the cases, contained a single GFP-CD63 molecule, while OE-EVs were predominantly multiple labeled. 

### 2.2. Intracellular Imaging with CRISPR/Cas9-Modified EVs

**Figure 4:**
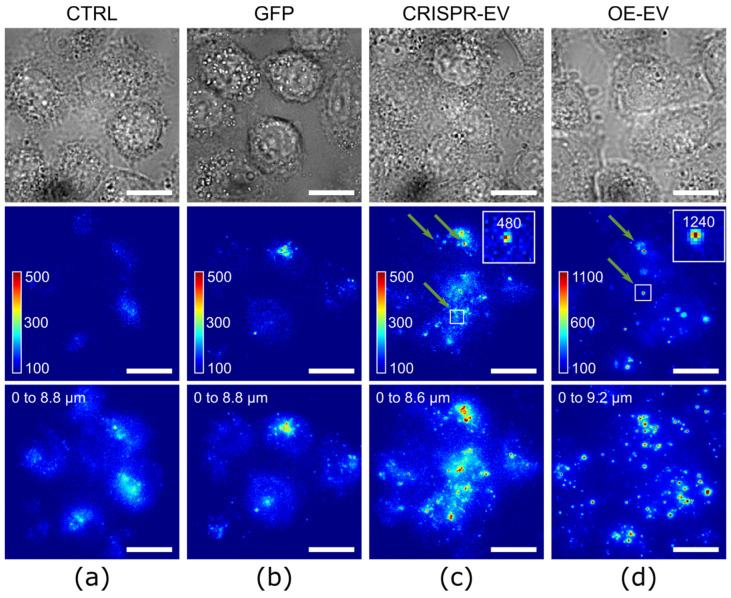
Intracellular imaging of GFP-labeled EVs. The HeLa cells were incubated with (**a**) PBS (CTRL), (**b**) recombinant GFP-protein (GFP), (**c**) CRISPR-EVs, or (**d**) OE-EVs for 1.5 h. The fluorescence images of one z-level (cross-section through the middle of the cell) are shown in the middle panel and the z-projections are shown in the lower panel. The arrows indicate the typical fluorescence signals of EVs. The scale bar is 10 µm.

To prove that the signal intensity of CRISPR-labeled EVs is suitable for intracellular localization studies, we analyzed the uptake of CRISPR-EVs and OE-EVs into HeLa cells. Figure 4 shows the transmitted light and fluorescence images of EVs after 1.5-h of incubation. Figure 4a represents the untreated control sample, showing the culture density (top) and auto-fluorescence (middle and bottom) of the cells. The middle panel represents a cross-section through the middle of the cell and the lower panel represents the z-projection. The control sample shows low auto-fluorescence of the cells, typically below a single GFP count rate. As a positive control, the cells were incubated with soluble GFP (Figure 4b). Here, we quantified the uptake of GFPs as they could potentially originate from disrupted EVs. The concentration (*p* = 10 µg/mL) of the soluble GFP was adjusted to match the fluorescence intensity of the OE-labeled EVs. As can be seen from Figure 4b (middle and lower panels), the soluble GFP was taken up by the cells and mostly accumulated in the proximity of the nucleus, thereby forming large clusters. This clustering is independent of the GFP concentration, as incubation with a concentration that is an order of magnitude smaller leads to the same effect (see Supplementary Figure S1b). Clustering is also confirmed by the comparison of intensity distributions (Supplementary Figure S2a) of GFP inside the cells with GFP on glass. Only 18% of the detected spots in the cells were single GFP molecules, the rest were agglomerations of two or more GFPs. In contrast to that, the CRISPR-labeled EVs that were taken up by the HeLa cells were more homogeneously distributed (Figure 4c). The images clearly show an enhanced uptake compared to the soluble GFP. The middle panel shows that the fluorescent signals which correspond to a single EV (indicated by arrows and a zoomed insert) are discernible in the HeLa cells. The results indicate that, on average, there are ~10 times more fluorescent spots per cell in the CRISPR-EVs-incubated cells than in the cells that were incubated with soluble GFP. A significantly higher signal was observed in cells that were incubated with OE-EVs (Figure 4d). Here, many bright peaks are visible in the HeLa cells. The zoomed insert shows a signal that most likely originates from an EV carrying multiple GFP-CD63 proteins. The projection image (bottom) shows localized bright spots within the cells which correspond to accumulated OE-EVs. 

Next, we performed a detailed statistical comparison of the intracellular fluorescent data that is analogous to the comparison of the EVs on glass. The results are summarized in Supplementary Figures S2 and S3. The fluorescent signal distributions of the CRISPR- and OE-labeled EVs which were taken up by the cells, shifted to higher values compared to their signals on the glass substrate (Supplementary Figure S2b,c). This indicates an aggregation of the EVs inside the cells in subcellular organelles. Using the same convolution procedure as used before (see results in Figure 3), one can estimate the number of aggregated EVs in one spot. For that, the fluorescence distribution that was obtained for CRISPR-EVs on glass was taken as ‘single-molecule fluorescence’ probability density (*P*_1_(*C*)) and used for the convolution of the fluorescence distribution of CRISPR-EVs in the cells (Supplementary Figure S2b). According to the performed analysis, only 23% of the detected fluorescent peaks were single EVs (or single GFP-CD63 constructs), around ~46% were twice as bright as single EV, and the rest were multiple EVs.

We have also compared the signal distributions of the CRISPR-labeled EVs inside of the cells to the signals of the soluble GFP on glass (Supplementary Figure S3a). Compared to the single-molecule signals, the results indicated that the CRISPR-labeled EVs showed multiple GFP signals in the HeLa cells. The statistical analysis showed that only 19% of the of the detected fluorescent peaks inside of cells corresponded to single-molecule GFP signals, ~38% to double, and ~4% to triple molecule GFP signal, respectively. The remaining detected fluorescent peaks (~39%) corresponded to four or more (≥4) GFP signals. 

A similar analysis was performed for the comparison of the OE-EVs in cells and GFP signal distributions on glass. The statistical comparison is shown in Supplementary Figure S3b and revealed that ~5% of all OE-EVs signals had count rates that were similar to the single GFP fluorophores, ~9% had twice that signal, and the rest 86% had triple or higher the intensity of single-molecule GFPs. Overall, all types of EVs, independent of the transfection method, showed aggregation inside the cells. However, the CRISPR-EVs carrying mainly only one GFP fluorophore (83%), allowed a more quantitative imaging inside the cells. 

**Figure 5:**
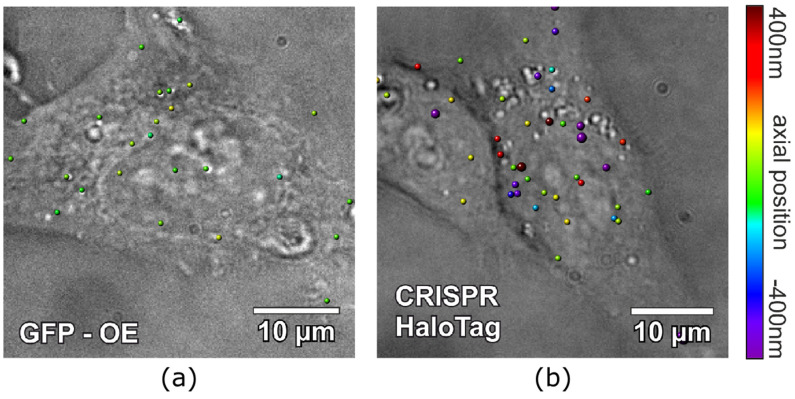
An overlay of EV localizations and brightfield image of the HeLa cells that were incubated with EVs. The colored dots represent the 3D localized position of EVs inside the cells. The diameter corresponds to the lateral position accuracy (multiplied by three for better visibility) of the localized EVs, the color corresponds to their z-position. (**a**) shows the 3D localizations of OE GFP-CD63 EV. (**b**) shows the 3D localizations of the CRISPR/Cas9-modified HaloTag^®^ EVs that were labeled with JF646.

The CRISPR/Cas9 system allows one to tag endogenous proteins not only with GFP, but also with other molecules. To prove the system flexibility we tagged using HaloTag^®^, which provides a high flexibility in fluorescent labeling due to its unique coupling mechanism of HaloTag^®^ ligand-tagged fluorophores [46,47,48,49]. In our case, we used a silicon rhodamine (SiR)-based JaneliaFluor^®^646 (JF646) dye coupled to a HaloTag^®^ ligand. The membrane-permeable dye provides ~3 times better average signal compared to a single GFP fluorophore (157 ± 65 counts/pixel for a single GFP (t_ill_ = 5 ms, *n* = 247, I ~ 3.2 kW/cm^2^), 1445 ± 835 counts/pixel for a single JF646 on average (t_ill_ = 10 ms, *n* = 1468, I ~ 4.4 kW/cm^2^). The signals were obtained from sparsely distributed dyes that were attached to a glass slide and the slightly different imaging parameters were taken into account by normalization (linear dependence between the fluorescence intensity and time/intensity was assumed). Consequently, the HeLa cells were incubated for 30-min with the JF646-labeled CRISPR-EVs and the GFP-labeled OE-EVs (for comparison). Afterwards, the cells were fixed and imaged at the single-molecule level. For the visualization of the EV distribution inside a cell, 3D reconstruction of astigmatism distorted single-molecule emitter point spread functions was used [54,56,57,59,60]. A high signal–noise ratio (on average >30) enabled the 3D localization of the subdiffractional EVs and aggregates inside the cells. The determined average localization precision in the cells was 35 nm lateral and 54 nm axial for the GFP-OE-EVs and 41 nm lateral and 69 nm axial for the JF646-CRISP-EVs, respectively. Figure 5a depicts an overlay of the brightfield image of the cells with the 3D reconstructed image of GFP-labeled OE-EV (colored dots). The dot color corresponds to the EVs axial positions. Similar 3D localization results were obtained with JF646-labeled CRISPR-HaloTag-EVs (Figure 5b). These EVs tended to have a more endogenous concentration of the CD63 in the membrane. A detailed comparison of the intensity distributions of single JF646 fluorophores to the distribution of JF646-labeled CRISPR-HaloTag-EVs proves this assumption (Supplementary Figure S4). Interestingly, despite the better signal–noise ratio of the JF646 fluorophore compared to single GFP, the axial position accuracy of the JF646-labeled EVs was slightly worse compared to the GFP-labeled OE-EV. This was caused by the bright EV signal of the OE-EV due to a huge fraction of the EVs carrying multiple CD63-GFP constructs.

## 3. Discussion

For the first time, we used the CRISPR/Cas9 method to tag the endogenous EV marker, CD63, with a fluorescent protein. We demonstrated the feasibility of this method and compared the isolated EVs on the single-particle level to samples derived from GFP-CD63 over-expressing cells. CRISPR-EVs avoid the potential over-expression artefacts and offer the possibility to follow unperturbed EVs after their up-take into cells. To compare the different labeling strategies, we quantitatively analyzed the fluorescence intensities of single EVs that were attached to a glass surface with single-fluorophore sensitivity. Strikingly, the analysis yielded a large fraction of single GFP-labeled EVs in the CRISPR EVs. This indicates a more endogenous level of proteins in the EVs that were isolated from CRISPR/Cas9-modified cells, leading to homogenously labeled EVs. Compared to a recently published study by Corso et al. [23], where fluorescence correlation spectroscopy (FCS) was used to determine the number of fluorophores per particle, our results indicate a lower number of fluorophores per EV, even in the over-expressing EVs. This discrepancy is likely because transiently transfected cells were used in their study to isolate the EVs, and, therefore, the over-expression of the fusion proteins was higher than in our stable cell lines. Our measurements are single-particle measurements and indicate that a large fraction of single- and double-labeled EVs are contained in EV preparations from stable cell lines. 

To evaluate our EVs for intracellular studies, we have demonstrated that EVs are internalized by cells more efficiently as compared to soluble GFP. Soluble GFP is taken up by fluid phase endocytosis, a slow and nonspecific mechanism whereby the cell constantly samples its environment. The more efficient up-take of the labeled EVs by cells indicates a more specific up-take mechanism, for example a receptor-mediated process [61,62,63]. Using the same analysis algorithm for the isolated EVs on glass, we consequently observed distributions and probability density functions that shifted towards multiple GFP signals in cells, indicating particle accumulation after internalization. Despite their lower labeling density, CRISPR-EVs can be detected and discriminated from auto-fluorescence after their up-take into cells. Generally, our results indicate that cellular over-expression of GFP-CD63, as shown with flow cytometry results, correlates with the number of fluorophores per single EV particle.

As a more flexible strategy to label EVs for imaging studies, we additionally used the CRISPR/Cas9 method with the HaloTag^®^ that was fused to CD63. This tag allows a post-isolation labeling with different fluorophores which can overcome the limitations that are associated with the use of fluorescent proteins such as GFP, especially for super-resolution microscopy. 

Localization microscopy imaging of the GFP-OE-EVs and the HaloTag^®^-modified CRISPR-EVs proves that 3D position reconstruction of the EVs in cells with localization precisions down to 41 nm lateral and 69 nm axial is granted. Due to the implemented CRISPR/Cas9 modification with the endogenous levels of proteins in the EVs with GFP or HaloTag^®^, true single-molecule fluorescence studies have been enabled. The results on intensity comparison indicate that JF646 dyes might aggregate inside the EVs compared to fluorophores on the glass slide (see Supplementary Figure S4c). 

With the use of fluorescent proteins and tags such as the HaloTag^®^ that was used in our study, our work offers a feasible and flexible labeling strategy for studying EVs. Hence, for the future application, 3D two-color localization microscopy can be used to co-localize the EVs with other vesicular particles inside the cells to further decipher the up-take pathways in the cells. The single-molecule methodology that was described in our work allows tracking of the EVs independently of the labeling ratio.

## 4. Materials and Methods

### 4.1. Transfections and Cell Culture

HEK293T cells (ATCC #CRL-3216) were cultured in Dulbecco’s modified eagles medium (DMEM, Thermo-Fisher, Waltham, MA, USA), high glucose, that was supplemented with 10% fetal bovine serum (FBS, Thermo-Fisher) and 1% penicillin/streptomycin (complete medium) at 37 °C in a 5% CO_2_ atmosphere. To generate GFP (and HaloTag^®^) CD63-expressing cells with the CRISPR/Cas9 technology, HEK293T cells were cultivated and seeded (~300,000 cells) in 12-well plates in DMEM-C. Two hours before transfection, the medium was changed to antibiotic-free medium (OptiMEM^®^ that was supplemented with 5% FCS; Gibco; Carlsbad, CA, USA). The crRNA:tracrRNA (guideRNA) complex was prepared according to the protocol of IDT (Integrated DNA Technologies, Coralville, IA USA) by mixing crRNA and tracrRNA with a Nuclease-free Duplex Buffer and heating the mixture to 95 °C for 5-min to a final gRNA concentration of 3 µM (guideRNA sequences are shown in Table 1). Transfection was performed using ScreenFect siRNA reagent by adding: 0.5 µg of Cas9 expression plasmid pX459 (Addgene #48139) containing a puromycin selectable marker that was separated from the Cas9 open reading frame (ORF) by a self-cleaving peptide, 0.5 µm of the GFP-CD63 HDR plasmid (or the HaloTag_CD63 HDR plasmid), and 30 pmol guideRNA duplex simultaneously to the cells. After 24-h, the medium was changed to DMEM-C and puromycin selection for transient uptake of pX459 was started after 48-h for two days. The cells were then expanded for flow cytometric analyses and cloning in complete medium without puromycin. The GFP-positive (GFP+)-sorted cells (or cells expressing HaloTag^®^) were diluted with complete growth medium and cultivated in 10 cm culture dishes (Thermo Fisher, Waltham, MA, USA) until single-cell derived colonies were clearly visible by the naked eye. The single colonies were trypsinized using cloning rings that were sealed to the dishes with sterilized vacuum grease, transferred to 48-well plates, and expanded to 6-well plates prior to freezing and further analysis. Transfection with the GFP-CD63 plasmid (Addgene #62964) for over-expression was performed with 1 µg of DNA and Endofectin Max reagent (Genecopoeia, Rockwell, MD, USA) according to the manufacturer’s intstruction. After cultivation of the transfected cells for several weeks, the GFP+ cells were sorted by fluorescence-activated cell sorting (FACS), cloned, and characterized as outlined above. The HeLa cells (#ACC57, DSMZ Braunschweig, Germany) were cultivated in DMEM high glucose with Glutamax (Gibco, Carlsbad, CA, USA) that was supplemented with 10% fetal bovine serum (FBS, Gibco) and 1% penicillin/streptomycin at 37 °C in a 5% CO_2_ atmosphere. 

### 4.2. Flow Cytometry and Cell Sorting 

The cells were trypsinized, washed once with phosphate-buffered saline (PBS), and resuspended in FACS buffer (PBS, 0.1% BSA, 2 mM EDTA). The flow cytometric analyses were performed with a FACSAria I cell sorter (Becton Dickinson, Heidelberg, Germany). The green fluorescent cells were detected in the fluorescein isothiocyanante (FITC) channel. The untransfected (parental) HEK293T cells were used to discriminate the GFP (and JF646) signal from autofluorescence. To enrich the GFP+ cells, a sorting gate was chosen in a FITC vs. SSC dot plot. The cell doublets were excluded in a FSC-A vs. FSC-W gate. The HaloTag^®^_CD63 cells were labeled with 250 nM JaneliaFluor^®^646 HaloTag^®^ Ligand (JF646; Promega, Madison, WI, USA) for 30-min at 37 °C. To enrich the JF646+ cells, a sorting gate was chosen in an APC vs. SSC dot plot. The sorting was performed in “Purity” mode, and the sorted cells were collected in complete medium and were either directly seeded at a low density for cloning or at a high density for further expansion before cloning. 

### 4.3. Integration-Specific PCR

The integration of GFP and the HaloTag^®^ in the genomic CD63 sequence of the cells was analyzed by PCR. Genomic DNA was isolated from single-cell clones using the Wizard SV Genomic DNA Purification Kit (Promega, Madison, WI, USA) according to the manufacturer’s instructions. PCR was performed with GFP- or HaloTag-specific forward primers and a CD63-specific reverse primer binding outside of the CD63 homology arm clones into the HDR plasmid. The primer sequences were designed with SnapGene software (from Insightful Science; available at snapgene.com) and ordered from IDT (Integrated DNA Technologies, Coralville, IA, USA) and are shown in Table 1. The PCR was performed using the High Fidelity Master Mix Q5 (New England Biolabs, Ipswich, MA, USA) according to manufacturer’s protocol on a CFX96 RealTime System C1000 (Bio-Rad, Hercules, CA, USA). The PCR products were separated by agarose gel electrophoresis and stained with ethidium bromide (Carl Roth GmbH, Karlsruhe, Germany) that was added directly to a 1% agarose gel. As a size control, the E-Gel 1 kb Plus Express DNA Ladder (Thermo Fisher Scientific, Waltham, MA, USA) was used and the gel was visualized with the Lourmat Fusion-SL-3500 WL imager (Vilber, Collégien, France). 

### 4.4. EV Isolation

Parental, CRISPR-GFP-CD63, or OE-GFP-CD63 HEK293 cells were cultured to a sub-confluent state in DMEM that was supplemented with 10% FCS and 1% penicillin/streptomycin. A 20% FCS solution was centrifuged at 110,000× *g* for 12-h to deplete EVs. For collection of the conditioned media, the media was changed to OptiMEM (Gibco, Carlsbad, CA, USA) containing 2% EV-depleted FCS, and supernatants of approximately 130 × 10^6^ cells were collected after 24-h. The cell number and viability of the EV-producing cells was verified with a CASY cell counter (OMNI Life Science, Bremen, Germany). The cellular debris, apoptotic bodies, and larger EVs were removed from the supernatants by centrifugation at 200× *g* (5-min), 2000× *g* (10-min) and 10,000× *g* (30-min) at 4 °C. The smaller EVs were obtained by ultra-centrifugation at 110,000× *g* (1-h 10-min) at 4 °C (Sorvall MX150, Thermo Fisher Scientific, Waltham, MA, USA) in a fixed angle rotor (Hitachi S58A-0095, Chiyoda, Japan). The resultant pellet was washed in PBS an additional time, concentrated by ultracentrifugation, and subsequently resuspended in 30 µL of PBS. The samples were stored at −80 °C. The protocol was adapted from Bobrie et al. [53].

### 4.5. Western Blotting

The isolated EVs and their corresponding EV-producing cells were lysed in RIPA buffer (25 mM TrisHCl pH 8.0, 150 mM NaCl, 1% Triton X 100, 0.5% Sodium Deoxycholate, 0.1% Sodium Dodecylsulfate (SDS)) containing protease inhibitors (Sigma-Aldrich, St. Louis, MO, USA). The protein concentration was analyzed via a Micro BCA Protein Assay kit (Pierce, Rockford, IL, USA). To confirm the successful isolation of EVs of endosomal origin, 2 µg of protein were separated in a 10% SDS-PAGE gel under non-reducing conditions for the detection of tetraspanins, and under reducing conditions for all other proteins. Following blotting to a PVDF membrane, the expression of the common EV markers were analysed using antibodies against CD63 (Merck, Darmstadt, Germany), CD81, TSG101, and Alix—with GM130 serving as a negative marker (all antibodies were purchased from Santa Cruz Biotechnology, Dallas, TX, USA). For comparison of the cellular GFP-CD63 expression by the CRISPR and OE cells, 20 µg of protein and antibodies against the loading control β-Actin (Santa Cruz Biotechnology, Dallas, TX, USA) were used.

### 4.6. High-Speed AFM Imaging

High-speed AFM (HS-AFM) [64,65,66,67] (RIBM, Japan) was conducted in tapping mode at room temperature (RT) with free amplitudes of 1.5–2.5 nm and amplitude set points that were larger than 90%. Silicon nitride cantilevers with electron-beam deposited tips (USC-F1.2-k0.15, Nanoworld AG), nominal spring constants of 0.15 Nm-1, resonance frequencies of ~ 500 kHz, and a quality factor of approximately 2 in liquids were used. For sample preparation, freshly cleaved mica was coated with 5 µg/mL Poly-D-Lysine (PDL) for 10-min and subsequently rinsed with PBS buffer. The isolated EVs were diluted 1:10/1:20 in PBS and incubated on the coated mica for 10-min, followed by careful washing with PBS. The samples were imaged in PBS.4.7. Fluorescence Spectroscopy

The fluorescence spectra of the undiluted EV samples or recombinant GFP-protein (a bacterially expressed and purified GFPmut2 variant, was a kind gift of J. Mairhofer, Engenes, Vienna, Austria) were acquired by excitation at 488 nm and detection of fluorescence emission between 510 and 600 nm on a Tecan plate reader (Tecan infinite M200 Pro, Tecan Group, Männedorf, Switzerland).

### 4.7. Fluorescence Microscopy

Confocal microscopy: Untransfected, CRISPR-GFP-CD63, or OE-GFP-CD63 HEK293 cells were seeded in Lab Tek 8-well chambers onto poly-D-lysine-coated glass coverslips. The nuclei were counter-stained with 1 µg/mL Hoechst 33342 dye (Sigma-Aldrich). Imaging was performed using a confocal microscope (FluoviewFV10i, Olympus, Vienna, Austria) using a 60x/1 NA water-immersion objective. the glass coverslips (No. 1, 0.16 mm) were cleaned by applying two cycles of 15min. of oxygen plasma and 15-min. of argon plasma (Zepto, Diener, Ebhausen, Germany) followed by coating with 0.1 mg/mL poly-D-lysine (Advanced BioMatrix, Carlsbad, CA, USA) for 20 min. The diluted EVs were adsorbed for 45-min. at room temperature and the unbound EVs were removed by four PBS washes. Imaging was performed on a modified Olympus IX81 inverted epifluorescence microscope with an oil-immersion objective lens (UApo N 100x/1.49 NA, Olympus, Vienna, Austria) [60,68]. A tube-lens with an additional magnification of 1.6 was used to achieve a final imaging magnification of 160 (corresponding to a pixel size of 100 nm). The sample was positioned with nanometer precision on a XYZ piezo stage (P-733.3DD, Physical Instruments) on top of a mechanical stage with a range of 1 × 1 cm that was adjusted by precision screws (TAO, JPK Instruments, Berlin, Germany). The fluorescence signal was detected using an Andor iXonEM+ 897 (back-illuminated) EMCCD camera (16 μm pixel size). The EVs were exposed to a solid-state laser with a wavelength of 640 nm (diode-pumped, Toptica Photonics, Graefelfing, Germany), a 488 nm solid-state laser (diode-pumped, Toptica Photonics, Graefelfing, Germany) and a 404 nm laser light (diode-pumped, Toptica Photonics, Graefelfing, Germany). The following filter sets were used: dichroic filter (ZT405/488/561/640rpc, Chroma, Olching, Germany), emission filter (446/523/600/677 nm BrightLine quad-band band-pass filter, Semrock, Rochester, NY, USA), an additional emission filter 525/50 M (Chroma Technology GmbH, Olching, Germany), and 700/75 M (Chroma Technology GmbH, Olching, Germany). For 3D measurements, a cylindrical lens (f = 1000 mm, Thorlabs, Newton, NJ, USA) was placed into the emission pathway between the microscope and the camera.

### 4.8. Intracellular EV Uptake

For the visualisation of the cellular EV uptake, the HeLa cells were seeded in Lab Tek 8-well chambers that were adhered to poly-D-lysine-coated glass slides. Once the cells reached 80–90% confluence, they were washed with PBS and the media was changed to low fluorescent FluoroBrite media (Gibco, Carlsbad, CA, USA) for 2 h. CRISPR or OE EVs were diluted 1:10 in FluoroBrite (that was supplemented with 2% EV-depleted FCS and 1% penicillin/streptomycin) and incubated with the HeLa cells for 1.5 h at 37 °C in a 5% CO_2_ atmosphere. To show that the unbound extravesicular GFP was not efficiently internalized, the cells were incubated with recombinant GFP protein and its concentration equalling the fluorescence intensity of CRISPR-EVs and OE-EVs as assessed by fluorescence spectroscopy. Extensive washing with PBS removed excess EVs/GFP. As a negative control, the EVs/GFP were substituted with PBS. The HaloTag^®^ EVs were diluted in FluoroBrite 1:10 (that was supplemented with 2% EV-depleted FCS and 1% penicillin/streptomycin) and labeled with 250 nM JaneliaFluor^®^646 HaloTag^®^ Ligand (JF646; Promega, Madison, WI, USA) for 30-min at 37 °C. The labeled EVs were then purified by OptiPrep™ (StemCell Technologies, Köln, Germany) density purification that was modified from Kowal et al. [6]. The labeled EVs were mixed with an equal volume of 60% OptiPrep density medium and overlaid with 30%, 20%, and 10% OptiPrep solution (that was diluted in 250 mM sucrose, 10 mM Tris-HCl pH8, and 1 mM EDTA). The samples were centrifuged at 110,000× *g* and 4 °C for 90-min by ultracentrifugation (Sorvall MX150, Thermo Fisher Scientific, Waltham, MA, USA) in a fixed angle rotor (Hitachi S58A-0095, Chiyoda, Japan). A gradient of JF646 dye without EVs was included as a negative control and 9 fractions were collected after centrifugation. Each fraction was measured in a Tecan plate reader (Tecan infinite M200 Pro, Tecan Group, Männedorf, Switzerland), excitation at 646 nm, and emission 685 nm ± 20 nm. Typically, the EVs were recovered in fraction 2 and 3 of the gradient, while free JF646 fluorophore was detected from fraction 5 to 9. The labeled and purified EVs were then incubated with the HeLa cells for 1.5-h at 37 °C in a 5% CO_2_ atmosphere. The cells were fixed by incubation with 4% paraformaldehyde in PBS for 15-min. Wide-field laser microscopy was performed as described above. The samples were illuminated for 5 ms with 3.2 kW/cm^2^ laser intensity.

### 4.9. Image Analysis

The fluorescent spots were identified and analyzed by custom-written software in Qt/C++. The high-performance non-maximum suppression algorithm was adapted (with permission) from rapidSTORM [69] and used to approximate the positions of the single-molecules in each frame [70,71]. Each fit was performed in a window surrounding the proposed position. The point spread function (PSF) was fit using the symmetrical two-dimensional Gaussian error function model [72] and successively approximated by the trust-region Powell dogleg algorithm [73]. The resulting integrated signal intensity was used to generate a probability density function (PDF) of fluorescence intensities for CRISPR-EV, OE-EV, and GFP protein by dfittool of MatLab software (MathWorks, Natick, MA, USA). 

### 4.10. Probability Density Function Convolution

The probability density function convolution was based on theoretical work according to Schmidt et al. (1996) [58,74]. Starting with the probability density *P*_1_(*C*) for the counts *C* from a sample with a very high probability that each fluorescent spot represents only one fluorescently labeled CD63 in an EV, one extrapolates this probability density *P*_1_(*C*) to a probability density *P*_2_(*C*) for the hypothetical situation where each spot contains exactly two fluorescently-labeled CD63 by performing the convolution

(1) $$P_{2}(C)=\int P_{1}(C^{\prime })\cdot P_{1}(C-C^{\prime })\;dC^{\prime },$$

and then iterates the process for the probability densities *P_n_*(*C*) where each spot contains exactly *n* fluorophores

(2) $$P_{n}(C)=\int P_{1}(C^{\prime })\cdot P_{n-1}(C-C^{\prime })\;dC^{\prime },$$

In the next step, one compares the weighted sum of all these probability densities with the empirical probability density *P_emp_*(*C*) of a sample where the number of GFP molecules per fluorescing spot is unknown

(3) $$P_{emp}(C)\approx \sum_{n}w_{n}\cdot P_{n}(C),$$

When a best fit of both is achieved, the weighting factors *w_n_* directly give the probability that a fluorescing spot contains exactly *n* GFP molecules. 

## Figures and Tables

**Table 1:** The gRNA and primer sequences for CRISPR/Cas9 approach and integration PCR.

**gRNA**	**Sequence [5′–3′]**	**PAM [5′–3′]**
gRNA#3	GAGGGCGGGGGGATTAAAAC	TGG
gRNA#4	CCGGCAGCCATGGCGGTGGA	AGG
**Primer**	**Sequence [5′–3′]**	
GFP 18 F	ATGGTGAGCAAGGGCGAG	
CD63 gDNA F	CCCTCTCCTGCGGGTAAAGA	
Halo integration_F	CCGCTGACTGAAGTCGAGATGG	
gDNA Exb 3_R	CCCACTGCACAGGCCTAAGAG	

## Data Availability

The data that support the findings of this study are available from the corresponding author upon reasonable request.

## Supplementary Materials

The following are available online at https://www.mdpi.com/article/10.3390/ijms23010282/s1.
