# Supplementary material for: CRISPR/Cas9 Genome Editing vs. Over-Expression for Fluorescent Extracellular Vesicle-Labeling: A Quantitative Analysis
Source: Int J Mol Sci. 2021 Dec 28;23(1):282. doi: 10.3390/ijms23010282 (PMC8745383; doi:10.3390/ijms23010282)
Supplement: Supplementary file 1 [file ijms-23-00282-s001.zip › ijms-1506082-supplementary.pdf]

International Journal of *Molecular Sciences*

Supplementary Materials

CRISPR/Cas9 Genome Editing vs Over-Expression for Fluorescent Extracellular Vesicle-Labelling: A Quantitative Analysis

Karin Strohmeier [1], Martina Hofmann [1], Fabian Hauser [1], Dmitry Sivun [1], Sujitha Puthukodan [1], Andreas Karner [1], Georg Sandner [2], Pol-Edern Le Renard [3], Jaroslaw Jacak [1,4] and Mario Mairhofer [1,†,*]

[1] Department of Medical Engineering and Applied Social Sciences, University of Applied Sciences Upper Austria, Garnisonstraße 21, 4020 Linz, Austria; karin.strohmeier@fh-linz.at (K.S.); martina.hofmann@fh-linz.at (M.H.); fabian.hauser@fh-linz.at (F.H.); dmitry.sivun@fh-linz.at (D.S.); sujitha.puthukodan@fh-linz.at (S.P.); andreas.karner@fh-linz.at (A.K.); jaroslaw.jacak@fh-linz.at (J.J.)
[2] Center of Excellence Food Technology and Nutrition, University of Applied Sciences Upper Austria, Stelzhamerstraße 23, 4600 Wels, Austria; georg.sandner@fh-wels.at
[3] Center of Advanced Bioanalysis GmbH, Gruberstraße 38, 4020 Linz, Austria; lerenard.pe@gmail.com
[4] Austrian Cluster for Tissue Regeneration, 1200 Vienna, Austria
* Correspondence: mario.mairhofer@jku.at
† Current address: Department of Hematology and Internal Oncology, Johannes Kepler University, Altenbergerstraße 69, 4020 Linz, Austria.

Supplementary Figures

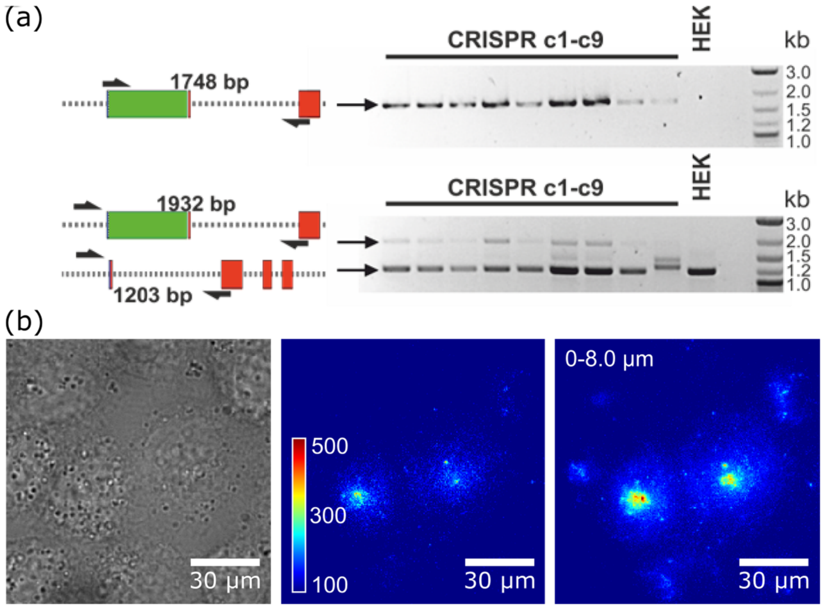

Supplementary Figure S1 (a) PCR analysis of CRISPR/Cas9 clones. Genomic DNA was isolated from nine single clones and was used as template for integration-specific PCR with a GFP-specific forward primer and a CD63-specific reverse primer binding outside of the CD63 homology arm cloned into the HDR plasmid (upper panel). All nine clones show correct integration of GFP at the endogenous CD63 locus, as demonstrated by the 1748 bp product, which is absent from parental HEK293T cells. PCR using a CD63-specific forward primer up-stream of the GFP integration site demonstrated that, most likely, a single CD63 allele was targeted in all clones, as the wild-type PCR product of 1203 bp is detectable together with the 1932 bp product including EGFP. (b) Intracellular imaging of HeLa cells incubated with recombinant GFP-protein (p = 1 µg/ml) for 1.5 hours. Fluorescence images of one z-level (cross-section through the middle of the cell) are shown in the middle panel and z-projections are shown in the right panel.

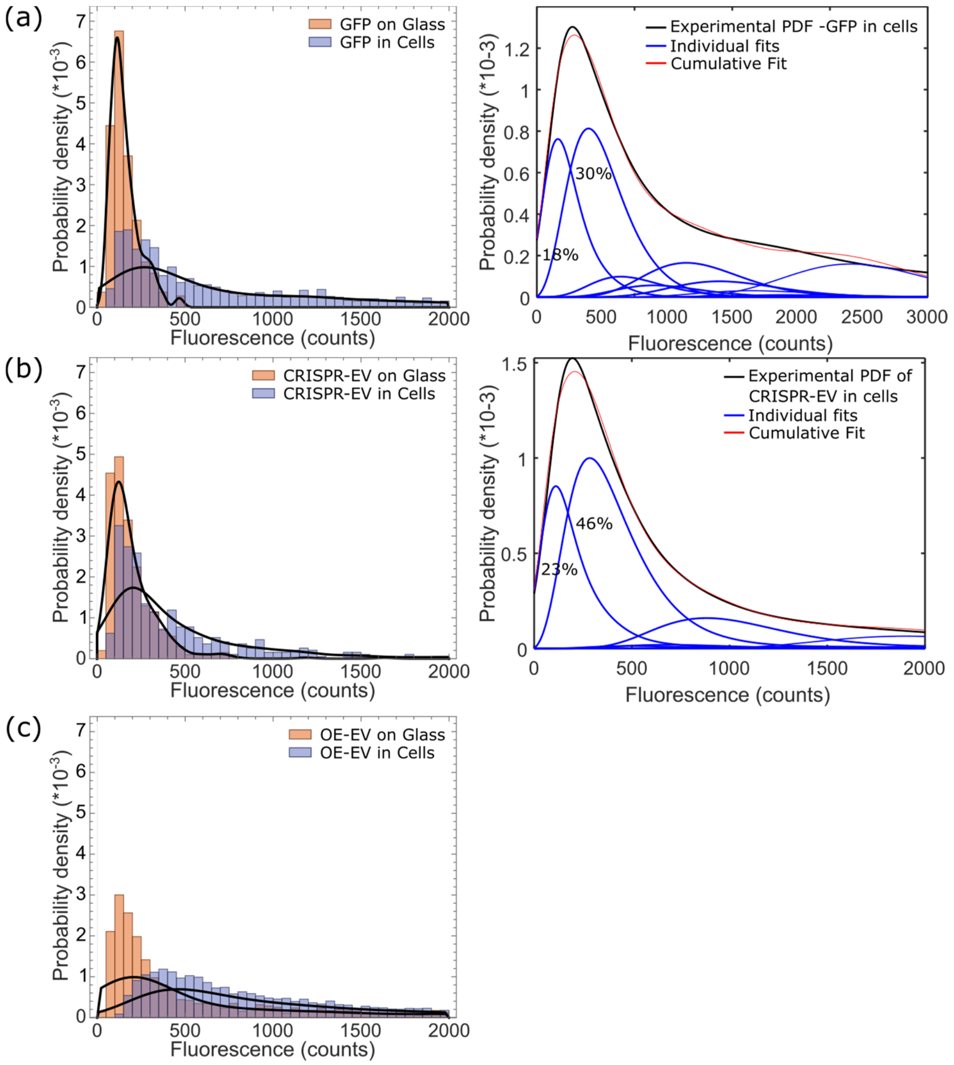

Supplementary Figure S2 Change of particle fluorescence through cellular internalization a) Comparison of GFP fluorescence immobilized on PDL-coated glass (orange) and internalized by HeLa cells (blue). The histograms are normalized and overlayed with their respective PDF (black solid lines). Right panel: PDF of GFPs internalized by HeLa cells is convoluted with multiple GFP PDFs immobilized on glass (18 %- DOL=1, 30 %-DOL=2, 5 %-DOL=3, 47 %-DOL ≥ 4). b) Comparison of CRISPR-EVs fluorescence immobilized on PDL-coated glass (orange) and internalized by HeLa cells (blue). Right panel: PDF of CRISPR-EV internalized by HeLa cells is convoluted with multiple CRISPR-EV PDFs immobilized on glass (23 %- DOL=1, 46 %-DOL=2, 1 %-DOL=3, 30 %-DOL ≥ 4). c) Comparison of OE-EVs fluorescence immobilized on PDL-coated glass (orange) and internalized by HeLa cells (blue). The histograms are normalized and overlayed with their respective PDF (black solid lines). (DOL – Degree of labelling).

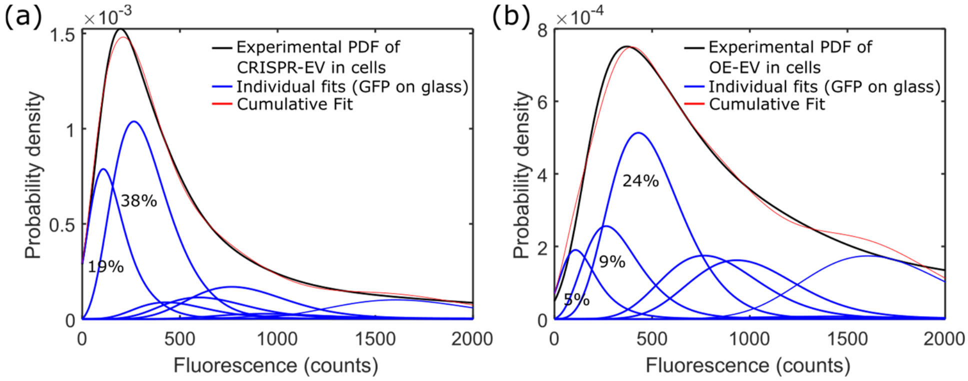

Supplementary Figure S3 PDF of a) CRISPR-EV and b) OE-EV internalized by HeLa cells (black curve) is convoluted with multiple GFP PDFs immobilized on glass (blue curves). CRISP-EVs: 19 %- DOL=1, 38 %-DOL=2, 4 %-DOL=3, 39 %-DOL ≥ 4. OE-EVs: 5 %-DOL=1, 9 %-DOL=2, 24 %-DOL=3, 62 %-DOL ≥ 4. (DOL – Degree of labelling).

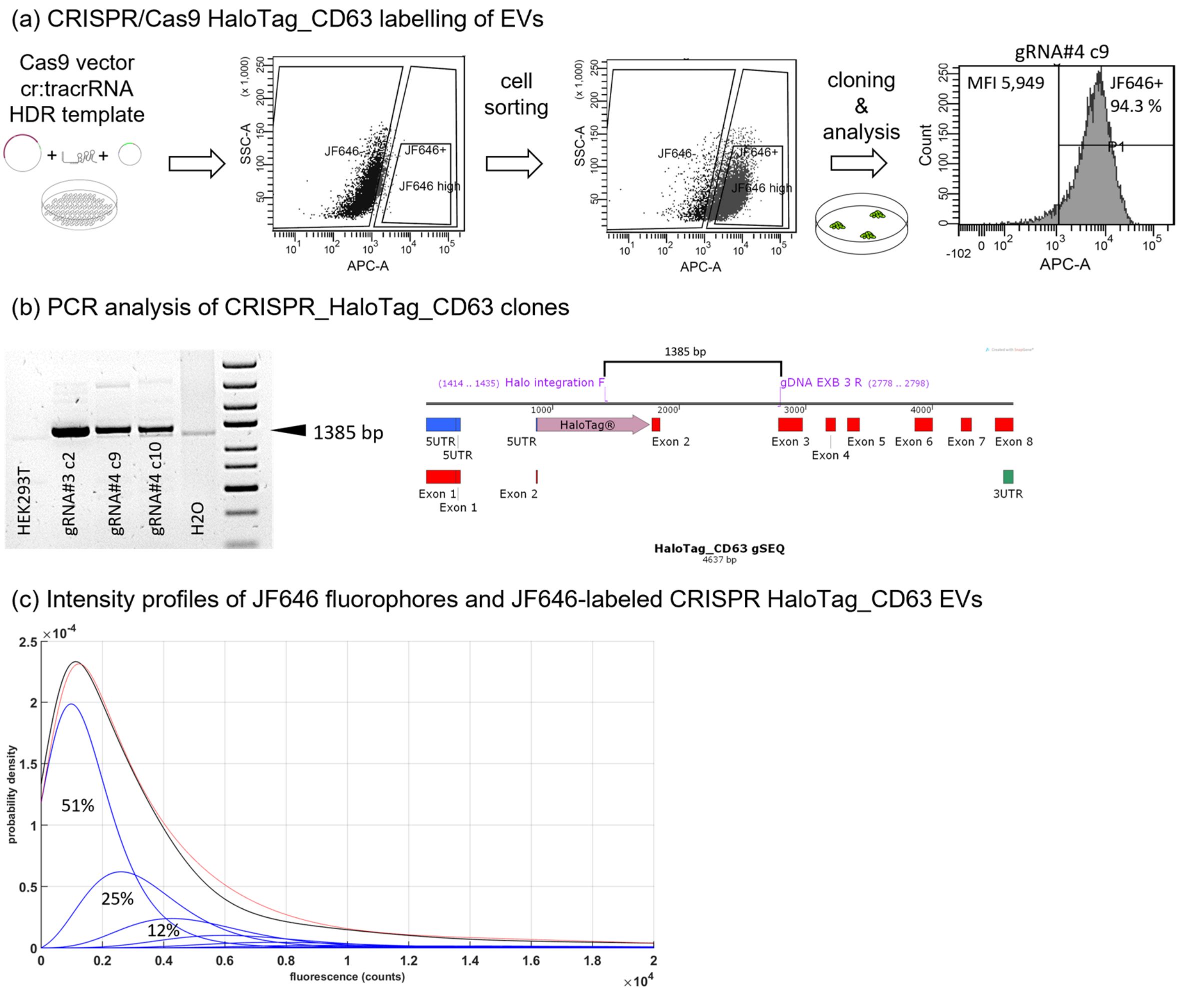

Supplementary Figure S4 (a) Schematic representation of HEK293T cell transfections. Cells were transfected with a Cas9 expression vector together with a guideRNA (cr:tracrRNA) targeting exon 2 of CD63 and a homology-dependent repair (HDR) template encoding the HaloTag® in-frame with the CD63 coding sequence. After transfection, the cells were labelled with a HaloTag® ligand JaneliaFluor 646 (JF646) for 30 min at 37°C and cells were enriched by FACS sorting. In the next step, single-cell derived clones were isolated and characterized by flow cytometry. (b) PCR analysis of CRISPR/Cas9 HaloTag®_CD63 clones. Genomic DNA was isolated from three single clones and was used as template for integration-specific PCR with a HaloTag-specific forward primer and a CD63-specific reverse primer binding site outside of the CD63 homology arm cloned into the HDR plasmid. All three clones show correct integration of the HaloTag® at the endogenous CD63 locus, as demonstrated by the 1385 bp PCR product, which is absent in parental HEK293T cells (control). (c) Comparison of the intensity profiles of pure HaloTag® ligand JF646 fluorophores and CRISPR HaloTag-CD63 EVs labeled with the ligand, both immobilized on PDL-coated glass. The probability density of single, immobilized JF646 signals is compared to the probability density of single or multiple JF646 labelled HaloTag® CRISPR-EVs. The comparison reveals that ~51 % are single JF646 signals (DOL=1), ~25 % were carrying two labels (DOL=2), ~12 % three (DOL=3), and rest were labelled with four or more fluorophores (DOL ≥ 4). (DOL = Degree of labelling).
